# Intermittent Right Ventricular Outflow Tract Capture due to Chronic Right Atrial Lead Dislodgement

**DOI:** 10.1016/s0972-6292(16)30779-3

**Published:** 2014-07-15

**Authors:** Partha Prateem Choudhury, Vivek Chaturvedi, Saibal Mukhopadhyay, Jamal Yusuf

**Affiliations:** Department of Cardiology, GB Pant Hospital, New Delhi, India

**Keywords:** Dual chamber pacemaker, lead dislodgement, Electrogram (EGM)

## Abstract

A 58 year old male, known case of type 2 diabetes and hypertension, had undergone implantation of a dual chamber pacemaker(DDDR) in 2007 for complaints of recurrent syncope and trifascicular block with a normal ejection fraction andnormal coronaries. His post implantation parameters were normal at that time.He now presented to our pacemaker clinic where his ECG done showed two types o fpaced complexes. The first few complexes were consistent with atrial sensed right ventricular apical pacing with left superior axis. Later complexes showed loss of atrial sensing with pacing from right ventricular outflow tract(inferior axis) with subtle oscillation in it's axis. On application of magnet, two pacemaker spikes were visible withinterspike interval of 120 ms and paced complexes with inferior axis starting from the first spike suggesting that the atrial lead was responsible for RVOT depolarization. On interrogation of the pacemaker, atrial EGM showed sensed activity from atrium followed by large sensed ventricular complex. Fluoroscopy confirmed that the atrial lead was dislodged and was intermittently prolapsing into the RVOT. Since the patient was asymptomatic, he refused any intervention and subsequentlyhis atrial lead was switched off by telemetry. The above case signifies that asymptomatic lead dislodgement is no talways manifested as loss of capture and even subtle variation of the axis o fthe paced complexes can provide us with a clue that can be confirmed by telemetry of the pacemaker and fluoroscopy.

## Introduction

**Figure 1:**
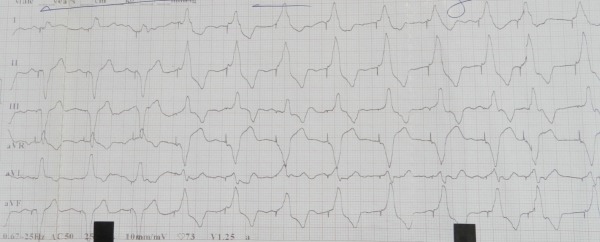
Baseline ECG showing spontaneous paced QRS complexes. The ECG shows paced QRS complexes at an approximate rate of 75 bpm. The first three complexes show p waves followed by paced QRS complex with left superior axis consistent with atrial sensed right ventricular apical pacing. The subsequent complexes do not show any p waves and have paced complexes with an inferior axis; within these complexes itself the sixth, seventh, tenth and eleventh complex show subtle widening of QRS and leftward shift in axis (aVL positive) as compared to fourth, fifth, eighth, and ninth complexes (aVL equiphasic). Only one pacemaker spike is seen and no clear p wave is made out.

A 58 year man presented to our pacemaker clinic for a routine follow-up visit. Previously, he had undergone implantation of a dual chamber pacemaker (DDDR) in 2007 (both leads tined) for complaints of recurrent syncope and trifascicular block (right bundle branch block with left atrial hemi-block and prolonged PR interval) on electrocardiogram. His left ventricular ejection fraction and coronary angiogram were normal. His pacing parameters after implantation, and at his last visit 4 years post-implantation, were both normal. He had been asymptomatic since then but on this visit, an interesting abnormality was recorded on the electrocardiogram (ECG) (Figure 1). What can be the cause for this ECG?

**Figure 2:**
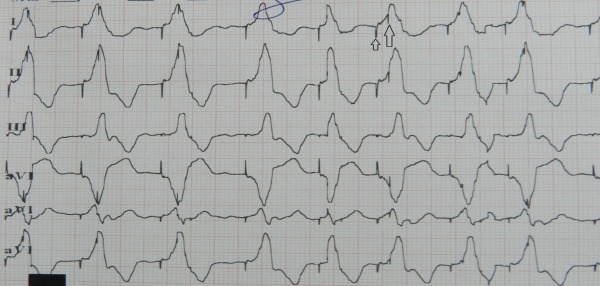
ECG with application of magnet after 4 spontaneous paced QRS complexes show two pacemaker spikes (small and large arrow) with interspike interval decreasing to 120 ms.

Thus in the initial complexes there is appropriate atrial sensed ventricular pacing proceeding from the RV apex while in the later complexes there is loss of atrial sensing with right ventricular pacing originating from the right ventricular outflow tract (RVOT) with subtle oscillations in the axis of paced QRS. On application of magnet, the following rhythm was noticed (Figure 2).

Before application of magnet, spontaneous pacing is seen with similar QRS morphology as the latter complexes in previous figure with no atrial sensing. However a pacemaker spike is seen buried within the first two QRS complexes with interspike interval of approximately 150 ms. On application of magnet the ventricular rate accelerates to 100bpm, the QRS width varies but morphology remains same, and the interspike interval decreases to 120 ms (small and large arrow). Thus on application of magnet, the ventricular depolarization is occurring from right ventricular outflow tract (RVOT) and presence of both the spikes with shortened interspike distance suggests that the atrial lead is responsible for this paced rhythm originating from the RVOT. As the initial complexes in Figure 1 suggest normal atrial sensing, the atrial lead is intermittently prolapsing into RVOT causing a change in paced QRS axis and the free lead movement is responsible for the subtle shifts in QRS width and axis seen within the RVOT paced complexes.

**Figure 3:**
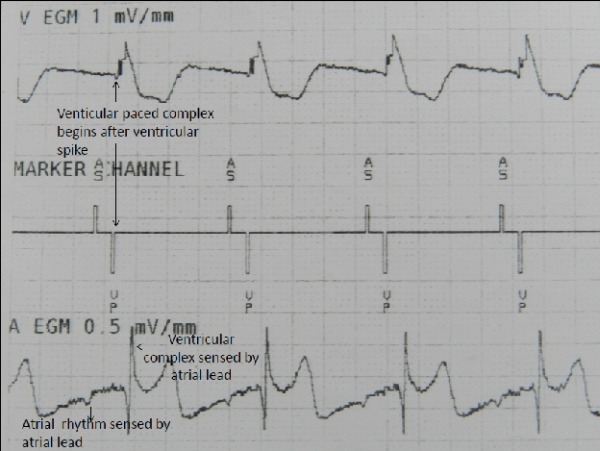
Pacemaker electrogram with top row showing ventricular EGM which shows paced ventricular complexes beginning after a ventricular pacing spike seen on marker channel. The atrial EGM at the bottom shows atrial activity being sensed by the atrial lead but large ventricular complexes are also recorded.

On interrogation of the pacemaker, atrial lead threshold and impedance were found to be high while the ventricular lead parameters were satisfactory. The sensed and paced AV delays of the pacemaker were set at 150 and 120 ms respectively. The following electrogram (EGM) trace was obtained (Figure 3). The top most EGM is from the ventricular lead and shows ventricular spike (coincident with Vp seen in marker channel in the middle) followed by paced ventricular complex. The bottom EGM is from the atrial lead and shows sensed activity from atrium (coincident with As seen in the marker channel in the middle). However it also shows a large ventricular EGM at the same time as the paced complex from the ventricular lead. This would indicate that during this interrogation, the atrial lead happened to be in lower atrium thus sensing atrial depolarization but was nevertheless close enough to apical ventricular lead to detect large ventricular EGM.

**Figure 4:**
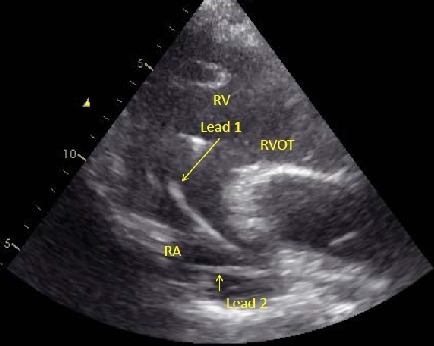
Parasternal short axis view on 2D echocardiogram showing lead 1 across the tricuspid valve into RV outflow while lead 2 is seen in RA.

**Figure 5:**
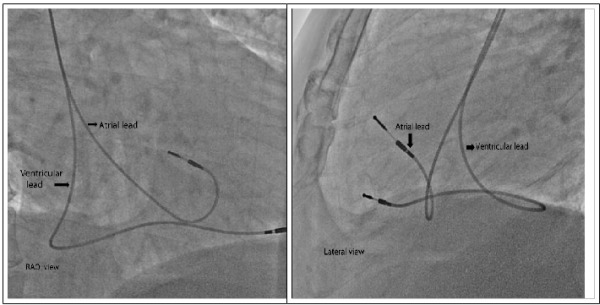
Fluoroscopy images in right anterior oblique 400 and lateral views show that the atrial lead is prolapsing into RVOT while the ventricular lead is in RV apex.

Fluoroscopy confirmed that the atrial lead was indeed dislodged and was intermittently prolapsing in the lower RVOT (Figure 5). A trans-thoracic echocardiogram showed normal left ventricular function and confirmed the fluoroscopic findings (Figure 4).

The ECG tracing in Figure 1 is thus explained by the run-away atrial lead which, dislodged from the right atrial appendage, is now intermittently prolapsing in the RVOT. Atrial sensed ventricular pacing originated from the original RV apical lead when the atrial lead was in right atrium; however when the atrial lead prolapsed in RVOT it lost atrial sensing and started pacing with an expected inferior axis. On application of magnet, only the paced rhythm from the atrial lead prolapsed in the RVOT was seen because the magnet caused asynchronous pacing from this lead. The pacemaker spike from the RV apical lead was seen after an expected 'AV delay' of 120 ms with no contribution to QRS complex. Since the atrial lead freely moved in the RVOT, there were subtle changes in paced QRS axis and width.

A delayed dislodgement of the atrial lead from the right atrial appendage was diagnosed and the patient was explained possible consequences and advised revision of atrial lead. However he refused on grounds of being asymptomatic and subsequently his atrial lead was switched off and pacemaker programmed to VVIR mode. At 3 months of telephonic follow up, patient did not report any symptoms.

## Discussion

In the current era, the rate of lead malposition and dislodgement in a dual chamber pacemaker is around 2% [1]. Lead dislodgement is usually picked up by sudden drop in the pulse rate by the patient or during routine follow up by ECG/pacemaker interrogation Diagnosis is suspected if there is failure to capture or undersensing on an electrocardiogram. Telemetry of the pacemaker usually reveal elevated thresholds and elevated or normal impedance. Chest X rays done in postero-anterior and lateral positions confirm the dislodgement. However dislodgement can also present uncommonly by other ECG manifestations, such as constant or intermittent right ventricular stimulation by the atrial lead, as was seen in our case. There are several possible causes for atrial stimulus coincident ventricular pacing complexes. These include direct and preferential activation of the right ventricular outflow tract by an atrial lead positioned at the anterior and medial right atrium [2]; an atrial lead positioned at the tricuspid annulus causing simultaneous capture of both chambers during atrial pacing (cross-stimulation) with high atrial output [3]; insulation break with resultant lead cross-talk; and, a dislodged atrial lead physically prolapsing into RVO intermittently [4], as was seen in our case also. Very rarely it can also be due to pseudo-pseudo fusion with atrial stimulus coincident with a ventricular ectopic beat.

Our case was made interesting by the startling ventricular pacing complexes with alternating opposite frontal axis on ECG. Absence of AV relationship during paced complexes from RVOT with inferior axis, subtle oscillations in axis during RVOT pacing, and changing interspike distances in this case suggested a mobile right atrial lead prolapsing into RVOT. That the lead was not permanently dislodged into RV was suggested by the initial complexes in Figure 1 with appropriate atrial sensed RV apical pacing as well as the AsVp seen during interrogation. While ventricular safety pacing (VSP) was turned on in this patient at a default value of 110 ms, no spike was seen at this duration. However a second spike was indeed seen buried within QRS complex at a interspike duration which changed with magnet application. This suggests that either the initial depolarization from the prolapsed atrial lead was not sensed by the RV apical lead due to refractoriness or that VSP did not get activated due to underlying right bundle branch block with delay of impulse in reaching RV apex. Consequently the RV lead spike was seen at the expected 'AV delay' interval.

In conclusion, we report an interesting case of dislodged atrial lead with intermittent prolapse into the RVOT that presented with an uncommon ECG finding of alternate superior and inferior frontal axis of RV pacing.

## References

[R1] Eberhardt F (2005). Long term complications in single and dual chamber pacing are influenced by surgical experience and patient morbidity. Heart.

[R2] Pastori JD (2012). Intermittent capture of the right ventricular outflow tract by atrial pacing in a patient with a dual chamber pacemaker. J Electrocardiol.

[R3] Sela R (2012). Cross-stimulation due to malposition of an atrial lead of a dual-chamber pacemaker. Pacing Clin Electrophysiol.

[R4] Anderson MH (1990). Ventricular pacing from the atrial channel of a DDD pacemaker: a consequence of pacemaker twiddling?. Pacing Clin Electrophysiol.

