# Intermittent Right Ventricular Outflow Tract Capture due to Chronic Right Atrial Lead Dislodgement

**Published:** 2014-07-15

Address for Correspondence has been corrected as: Dr. Vivek Chaturvedi, Associate Professor, Department of Cardiology, GB Pant Hospital, New Delhi, India. E-Mail: chaturvedimd@gmail.com

